# Light people: Radha Nagarajan, Marvell CTO and NAE member

**DOI:** 10.1038/s41377-026-02305-6

**Published:** 2026-05-21

**Authors:** Yating Wan, Chenzi Guo

**Affiliations:** 1 Electrical and Computer Engineering, the Computer, Electrical and Mathematical Sciences and Engineering Division, King Abdullah University of Science and Technology, Thuwal, Saudi Arabia; 2 Changchun Institute of Optics, Fine Mechanics and Physics, Chinese Academy of Sciences, Changchun, China

**Keywords:** Semiconductor lasers, Integrated optics

**Figure:**
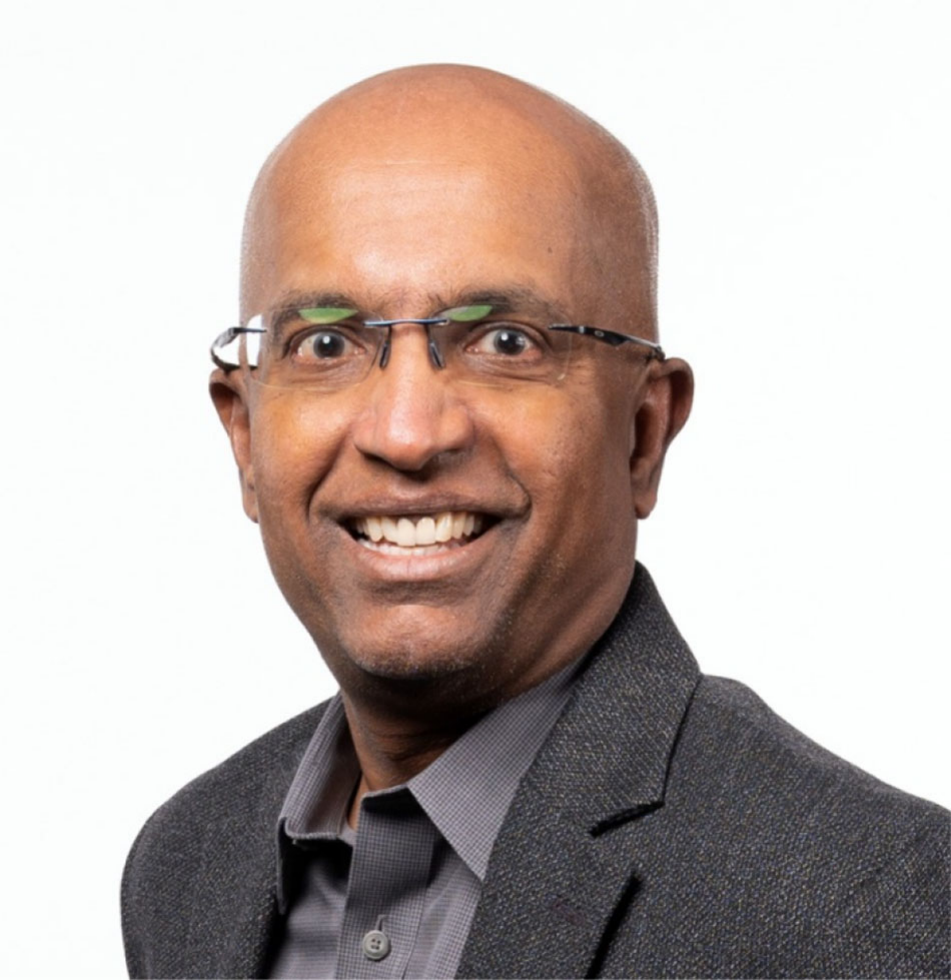

Short Bio of **Radha Nagarajan**. Dr. Nagarajan is currently the Senior Vice President and Chief Technology Officer of Marvell’s Optical Engineering Group. At Marvell, he manages the development of the company’s optical platform products and technology. Concurrently, he is a Visiting Professor at the Department of Electrical and Computer Engineering at the National University of Singapore. He received his B.Eng. from the National University of Singapore, M.Eng. from the University of Tokyo, and Ph.D. from the University of California, Santa Barbara, all in Electrical Engineering. Dr. Nagarajan has been elected to the National Academy of Engineering (US). His other recognitions include the IEEE/LEOS Aron Kressel Award, the IPRM Award and the OPTICA David Richardson Medal for breakthrough work in the development and manufacturing of photonic integrated circuits. He has been awarded more than 250 US patents. He is a Fellow of the IEEE, OPTICA, and IET.

**Q: You have worked across several companies, and your remarkable achievements have led to your election to US National Academy of Engineering. Through your career path, what dominates in your thinking about ‘what matters’ as integration scaled up?**

A: After my PhD with Prof. John Bowers, for the past 30 years, I’ve been working on photonic integration—first at Infinera, then Inphi, and now Marvell. If you look at these roles, they are a snapshot of how the industry has evolved. When I started at Infinera in 2001, our goal was to build photonic integrated circuits based on indium phosphide, primarily targeting long-haul applications. At that time, people were not talking about datacenter interconnects. Servers were still connected using conventional electrical cables, such as Cat5 or Cat6.

In 2013, I transitioned to Inphi and moved from indium phosphide to silicon photonics. While indium phosphide was already a mature platform, silicon photonics was emerging quickly. At that stage, our focus was still largely on applications outside the datacenters.

Later, when I moved to Marvell, the explosion of AI and HPC dramatically expanded the market. Silicon photonics is now being deployed at large scale—not only for outside the datacenter but also within the datacenter. My work during this period has been more centered on heterogeneous integration.

**Fig. 1:**
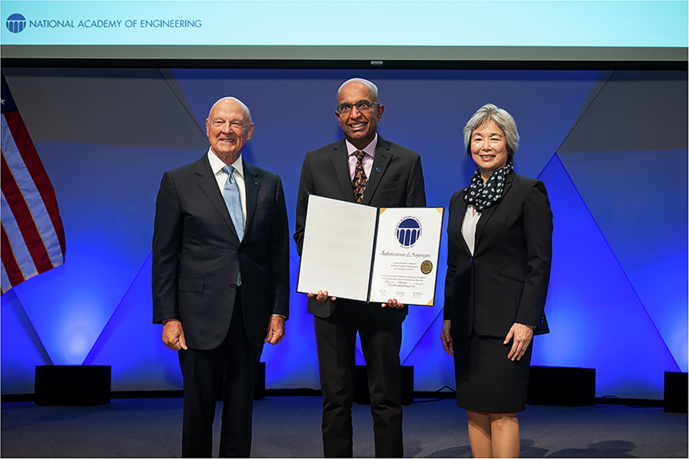
Dr. Radha Nagarajan elected to US National Academy of Engineering

**Fig. 2:**
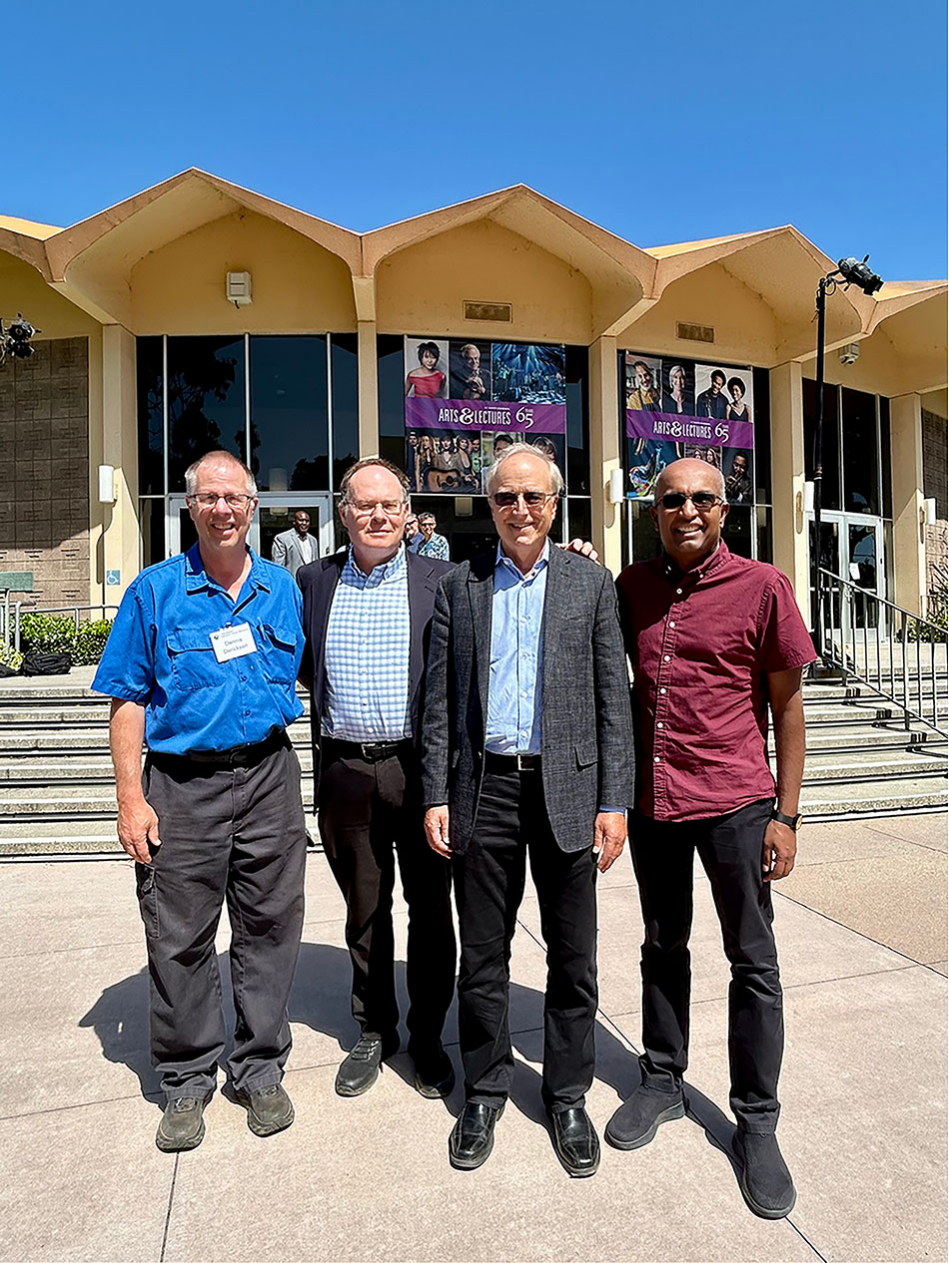
Dr. Radha Nagarajan was Prof. John Bowers’ first Ph.D. student to graduate, The photo was taken at John’s retirement party at UCSB, with two others who graduated subsequently

A common theme that has guided me in my career all along is turning advanced technology into a real product. This requires achieving large-scale manufacturing, maintaining yield at a profitable level, and ensuring reliable deployment in the field. These challenges are where we invested most of our effort, always with a strong focus on market needs. People often underestimate how much time, effort, and investment it takes to turn a promising technology into a commercial product, but this process has consistently been at the core of my work. (Figs. 1, 2)

**Q: At what point do you think silicon photonics will take over discrete components/modules?**

A: I would say silicon photonics has already taken over to a large extent, especially at 100 G and higher data rates. Yield is not something unique to just silicon photonics—it is a critical requirement for any practical technology. However, what silicon photonics uniquely enables is particularly important for the industry. First, it allows companies without their own foundry, like Marvell, to design and manufacture highly complex photonic components by leveraging the existing semiconductor infrastructure. Second, it provides true scalability. Marvell, for example, has been one of the well-known companies implementing silicon photonics for applications between datacenters. Intel pioneered silicon photonics for applications inside the datacenters. Today, many of the large transceiver companies have mostly transitioned to silicon photonics. Given this level of adoption, it is fair to say that silicon photonics has already ‘taken over’ in many key segments of the optical interconnect market.

**Q: Many silicon photonics systems still rely on external laser packaging. Do you see integrated light sources becoming mainstream, or will off-chip lasers dominate for practical reasons?**

A: If you look at the integrated laser work published over the years, including our early demonstrations, Intel’s results, and developments at Infinera (now Nokia), it is clear that integrating light sources heterogeneously on silicon or monolithically in InP is commercially viable. The real obstacle is not technical capability, but perception—many people still assume that integrated lasers will suffer from reliability issues. One concern stems from the behavior of indium phosphide lasers at elevated temperatures. It is true that performance can degrade at high temperatures, but when operated around a base temperature of roughly 70–75 °C, these lasers can already operate perfectly well. In fact, Intel has demonstrated strong reliability in this regime. The key is careful design. Temperature variations can be significant, and the resulting wavelength shifts must be properly managed. In practice, engineers have already developed effective strategies to handle these challenges. So, from a technical perspective, I do not see major roadblocks. The bigger challenge may be changing people’s mindset.

Let me also make a prediction. Cooling large-scale server infrastructure is becoming increasingly challenging. The industry is already transitioning from air cooling to liquid cooling, and some are exploring immersion cooling. These approaches can provide more stable and potentially lower operating temperatures, which could create an opportunity for integrated lasers as well.

**Q: How do you see the potential of heterogeneous integration?**

**Fig. 3:**
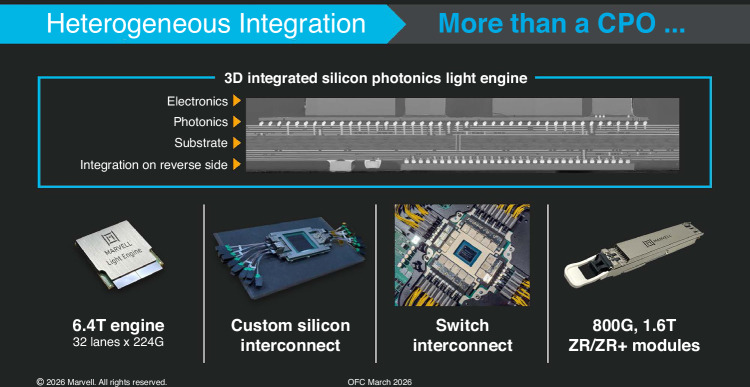
Heterogeneous integration

A: I think silicon photonics, as a platform for integration, has enormous potential. What we’ve achieved so far is only scratching the surface. There are multiple paths forward here. You can integrate different materials, combine multiple types of devices within the same material platform, or even move toward stacking multiple device layers. All of these approaches are actively being explored, and I believe many of them will enter production in the near future. As data rates continue to increase, heterogeneous integration will also involve incorporating new higher performance materials, opening up more possibilities. At the same time, integration itself is becoming more complex. We’re moving beyond traditional 2D integration. People are now talking about 2.5D, 3D, even 3.5D integration, which will create many new opportunities for system design and performance improvement. (Fig. 3)

**Q: You were one of the early voices pushing co-packaged optics (CPO) for AI systems, now more and more companies are doing this. What convinced you CPO was not just a research direction, but something the industry would truly need?**

A: The reason I have advocated for CPO stems from my firm belief that heterogeneous integration is the key to high-speed applications. During my time at Inphi (now at Marvell), we were already seeing the limits of traditional approaches. As data rates continued to increase, techniques like wire bonds simply could not keep up. The logical next step was to bring electronics and photonics closer through heterogeneous integration. That was the original motivation—overcoming speed and bandwidth bottlenecks. But as we developed these approaches, it became clear that they also align very well with what CPO is trying to achieve. Over time, the value of co-packaging has expanded beyond just pushing the highest data rates. We now see it being applied even at relatively lower data rates, especially in large-scale AI systems. Whether you look at scale-up, scale-out, or scale-across systems, they all benefit from tightly integrated photonics with electronics. So, in a way, heterogeneous integration is the essence, and CPO is one of its most important applications.

**Q: Speed is the reason that drives us from ‘pluggable’ to ‘on board’. Many believe ‘pluggable’ can be extended with better DSPs and packaging, while others argue they won’t scale forever’. From your perspective, will this ‘extension strategy’ scale indefinitely? If you had to point to a single limiting factor, what would ultimately block ‘pluggable’ from keeping up with 100** **T, or 200** **T switch ASICs?**

A: In my opinion, with 100 T or 200 T switches and systems at the horizon, you will need co-packaging. It’s true that the industry is very comfortable with ‘pluggable’ solutions, and there is a strong incentive to extend their life as much as possible. In many scale-up systems, copper will continue to be used as long as it makes sense from a power and cost perspective. For the same reasons, data center operators would naturally prefer to stay with pluggable solutions.

However, when developing new products, you have to look ahead. Since late last year, analysts have begun including CPO in their revenue projections, which signals a real shift in the industry. In fact, data center operators and hyperscalers are now actively funding CPO developments. That tells you this is no longer just a research direction. Technically, pluggables can be pushed further, even beyond 200 G per lane, but the cost is power. As you scale bandwidth, the power required for high-speed electrical interfaces and DSPs becomes increasingly difficult to manage.

This is where CPO becomes very attractive, especially for scale-up architecture like GPU or accelerator interconnects. I actually think the first major deployment for CPO will happen in scale-up systems, and then expand into scale-out.

We’re already seeing strong signals from the ecosystem. Nvidia has demonstrated multiple generations of CPO platforms, including at GTC and ISSCC. From a foundry perspective, this aligns very well with TSMC’s roadmap. The idea of tightly coupling electrical compute with optical I/O is no longer a dream—it could be just a year or two away.

That said, predicting the exact timing is always difficult. It’s much easier to explain the past than to forecast the future. When you work at the intersection of technology development and product deployment, there is always uncertainty about when the market will be ready. In many ways, developing new technologies can feel like placing a bet—and that’s simply part of innovation.

**Q: People often say that co-packaging is always the difficult part, what do you think about that?**

A: That perception largely comes from how optical packaging used to be done. If you compare it to electrical packaging, the difference is quite clear. Electrical packaging has been highly automated for a long time. But if you look at the optical packaging in early days, you’ll find yourself doing things one at a time, wire bonding electronics and manually aligning fibers, which are slow and not scalable. But once volume kicks in, things start to change. A lot of these processes are now highly automated. There are still operators involved, but the work is largely handled by automatic equipment. Efficient fiber coupling is still a challenge, no question. But there are already solutions out there, and they’re getting better. As volume continues to ramp, I believe the fiber coupling part will gradually be automated as well. If you look at where we are today, photonics manufacturing is already very different from five years ago, and nothing like ten years ago. And honestly, things are only going to move faster from here.

**Q: Are Through-Silicon Via (TSVs) essential for CPO?**

A: We demonstrated and published extensively on TSV as an option way to integrate electronics on top and then route down into silicon photonics bottom layer. But you don’t have to always do that. You can flip the architecture and put the optics on top instead, and some groups are actively exploring that. One reason people hesitate is thermal. Electronics tend to run hotter than optics, so it’s often easier to heat-sink from the top, if the electronics are there. But that’s not a fundamental limitation—there are many alternative thermal management strategies people are exploring as well.

There are also practical considerations in the fab that may lead to a preference for one configuration over another. Early on, there was a strong tendency to adopt TSV-based approach. For example, you now have packaging techniques like fan-out wafer-level packaging (FOWLP), which does not require TSVs at all. Several companies are already offering this technology, and some are even beginning to deploy CPO based on it.

So, the absence of TSVs is not a fundamental roadblock—there are multiple viable ways to implement these systems.

**Q: What are the expectations of the industry for the academia?**

A: Speaking from the industry side, I think there needs to be a balance. Once a technology that originally came out of universities starts being deployed at scale, that’s usually a good time for academia to move on to the next thing. Take co-packaging photonics as an example, it’s highly multi-disciplinary: material science, electrical engineering and mechanical engineering, all of them come together. There are still plenty of challenges. Yes, there are certain standard ways of doing packaging, but we’re always asking; are there new materials, new techniques that we’re missing? That’s where universities can really contribute—looking at directions that industry isn’t focusing on or simply doesn’t have the bandwidth to explore deeply.

On the contrary, the “speed game”, e.g., pushing performance to the limit or scaling something into a real product, usually belong to companies with the resources to do it. Academia sometimes tries to compete in that space, and occasionally it works, but most of the time it doesn’t. If you are competing directly at the product level, it’s probably not the best use of your time. An extreme example would be a university professor trying to build a full-scale foundry process. So, from an industry perspective, the expectation is simple: find synergies with the industry. Focus on what’s complementary, more fundamental, and more forward-looking—and help define what comes next.

**Q: You hold a visiting faculty position at the National university of Singapore. What’s the criterion when you hire a student?**

A: For students joining my group, the first requirement is clear: they must demonstrate that they can actually get things done. At the end of the day, execution matters. But beyond that, I really value a strong understanding of fundamentals. These days, with all the AI tools available, it’s very easy to get answers. In a way, that is no longer the hard part—almost anyone can do it. What really matters is whether you can dig into a problem from first principles and truly understand it. I’m not against tools—we rely on them to design complex systems. But tools are just tools. You still need to know what you’re doing. I appreciate students who can argue with me from first principles on why my suggestion may be wrong. If you can tell me where I’m missing the point, you’re hired. And what separates good graduates from a great graduates is, the latter actually understand and appreciate what they’ve done, not just that they’ve done it.

**Q: How to encourage the industry to publish more?**

A: I’ve always tried to publish, but it has not been easy—certainly not at the level I would have if I had stayed in academia. There are two main reasons. First, industry work is closely tied to company revenue, performance and competition, so there are always constraints on what you can share. Second, it’s really a matter of hours in the day. Most engineers are fully focused on their day-to-day work, and publishing becomes a lower priority. Nevertheless, I do think the industry should publish more. Both academia and industry benefit from understanding what the other side is doing. Currently, we are seeing more venues—especially conference proceedings—that strike a balance. They maintain academic rigor but also give industry some room to share insights without disclosing all the details. Journals and platforms that allow this kind of balanced disclosure are very valuable. And honestly, even conversations like this—interviews, discussions—are another way of publishing and sharing knowledge.

**Q: You have said that scaling AI workloads forces a rethink of where power is spent and how optics interfaces with compute. From your full-stack view across photonics, electronics, packaging, etc., what do you think people in our community still miss when they think about scaling photonics for AI in terms of power?**

A: I think people still underestimate how critical interconnect power is going to become. As data centers scale, you’re not just adding more compute—you’re adding a lot more interconnects. In fact, the real challenge today is no longer just making transistors smaller or faster but moving data efficiently—especially between processors and memory. That is really where the power bottleneck is. Nowadays, larger and larger data centers mean more and more interconnects, the number of interconnects can be ten to a hundred times larger than the number of XPUs deployed, depending on how they are connected. If they are connected in a full-mesh network, the number tends to be higher. Therefore, if you want full connectivity—especially beyond a single server, across racks—the limiting factor will sooner or later becomes the power consumed by the interconnects. And that is something you definitely don’t want. You do not want the interconnects to end up dominating the overall system power.

**Q: Currently, what percentage of the total power consumption is due to interconnects?**

A: It depends on how you define it. If you only count the pluggable interconnects between racks, that’s typically around 15%. However, if you also consider interconnects between XPUs, the cost of moving data becomes much more significant. I have seen estimates that go beyond 50%. So, the real number comes down to what you include and how you calculate it. Even within an XPU, there are interconnects, but if we set those aside and focus only on the connections between XPUs, you still need to account for power in copper traces, links to memory, etc.

**Q: Over the past 20 years, the energy per bit has dropped dramatically—by about a factor of 100—yet the total module power has still increased by roughly a factor of 10. How do you view this reality?**

A: Power only goes down if you stop computing. Otherwise, it is going to keep going up. Even if we make interconnects much more efficient—and we will—the savings just disappear. In practice, that power tends to get reinvested into making XPUs or memory more powerful. That is just how the system evolves. So, I don’t think the real question is whether we can reduce overall power consumption. The more relevant question is: are we using that power in the most efficient way? Some people would argue that processors are where the value is created, while interconnects are just supporting infrastructure. But if interconnects become the bottleneck then that assumption no longer holds.

**Q: What should be the priority factors of the systems?**

A: For AI applications, I would say the key factors are latency, power consumption, and data rate density, rather than raw data rate. Same with thermal dissipation, it is not about how much total power you’re dissipating, but how much power you’re dissipating per unit area. That is what really becomes challenging as systems scale. So, in many ways, the focus is shifting from absolute performance to how efficiently you can deliver that performance within tight power and thermal constraints.

**Q: When do you think the supply chain will be mature enough for CPO, silicon photonics, heterogeneous integration to really become the mainstream?**

A: I would say they are already among the mainstream technologies. At Marvell, for example, we are already building silicon photonics integrated platforms. The industrial capability is already there; what we really need now is volume. We have been pushing this forward for a long time, and the ecosystem has grown a lot. Compared to when we first started, it is a completely different landscape. There are now number of companies and foundries involved, with varying levels of capabilities. Even TSMC is investing heavily in co-packaging, alongside its core CMOS business. So, from both the technology and supply chain perspective, things have largely caught up. At this point, it is less about capability and more about adoption. We are really just waiting for large-scale deployment to fully kick in.

**Q: You once showed a ‘shoulder-to-shoulder’ architecture where the DSP sits very close to the silicon photonics engine. If we move toward even tighter integration, with a DSP-less optical engine where the host ASIC drives the photonics more directly, what co-design changes will become mandatory across the system?**

A: It really depends on the architecture and where you want to place the electronics. If you move the DSP further away, it can effectively sit inside the host ASIC. That is a scenario where we are today, where many of the DSP functionality has been absorbed into the host. If you think about the “shoulder-to-shoulder” example, the idea was to keep things close for performance and power reasons. But whether you keep a local DSP or not really comes down to system trade-offs, especially power consumption and what functions the customer needs. In some cases, you may still want a local DSP for specific features or signal processing. But strictly speaking, you don’t always need it. If the system can tolerate it, and the host can take over those functions, then a DSP-less approach becomes possible. So, the co-design challenge is really about partitioning—deciding where the various functions should live, how much power you’re willing to spend, and how tightly you want to integrate everything.

**Q: What changes have AI clusters brought to optical interconnects?**

A: Optical interconnects have been around for a long time, whereas AI clusters are a very recent driver. What they have really done is accelerate everything. They have pushed volumes up significantly and created an exponential growth in demand. Because of AI, we may reach a point where people become comfortable with electronic chips having optical I/O. Right now, it is still experimental. But if we look ahead for ten years, and AI continues to grow, while we start deploying switches and XPUs with optical I/O, that would be a real shift. It would fundamentally change how we think about optics—not just as a link between systems, but as something much more tightly integrated with compute. And I do believe that’s where we’re heading.

**Q: For young researchers and engineers who want to impact AI systems through photonics, what areas should they focus on, and how can they build the right skills?**

**Fig. 4:**
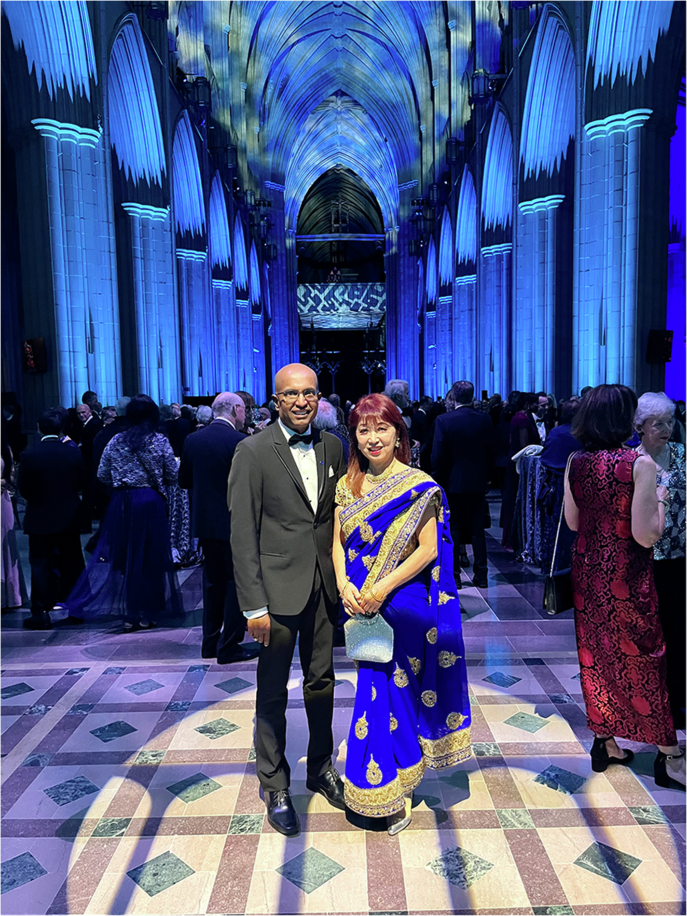
Dr. Radha Nagarajan and his wife at the induction ceremony to the National Academy of Engineering

A: Right now, we are in the middle of the AI revolution. But when I was a student, I had no idea photonics would become what it is today. So, my first suggestion is still to focus on fundamentals and to find what you are truly passionate about. If you decide that your passion lies in AI, then you need to think carefully about whether you want to work on hardware or software. Personally, I would suggest hardware—because, AI writes great software. But hardware is harder to replace. In terms of preparation, you need a strong foundation. Learn not just the algorithms, but also understand how to implement them efficiently. Build both breadth at the undergraduate level and depth at the graduate level. In the future, there will be increasing demand for optical circuit designers. Some universities are already offering courses that train students to understand the full photonics design flow. It is not just about running simulations like BPM or using numerical tools; it requires a much deeper understanding. In the end, fundamentals are what matter most. Whether you work in electronics, photonics, or AI, that foundation will carry you a long way. (Fig. 4).

